# Prognostic significance of prospectively detected bone marrow micrometastases in esophagogastric cancer: 10-year follow-up confirms prognostic significance

**DOI:** 10.1002/cam4.470

**Published:** 2015-04-27

**Authors:** Paul Ryan, Heidi Furlong, Conleth G Murphy, Finbarr O’Sullivan, Thomas N Walsh, Fergus Shanahan, Gerald C O’Sullivan

**Affiliations:** 1 Department of Pathology, Bon Secours HospitalCork, Ireland; 2 Cork Cancer Research Centre, University College CorkCork, Ireland; 3 Royal College of Surgeons of Ireland Department of Surgery, Connolly HospitalBlanchardstown, Dublin, Ireland; 4 Department of Oncology, Bon Secours HospitalCork, Ireland; 5 School of Mathematical Sciences/Statistics, University College CorkCork, Ireland; 6 Department of Medicine, University College CorkCork, Ireland; 7 Department of Surgery, University College CorkCork, Ireland

**Keywords:** 10-year follow-up, bone marrow micrometastases, esophagogastric cancer

## Abstract

We have previously reported that most patients with esophagogastric cancer (EGC) undergoing potentially curative resections have bone marrow micrometastases (BMM). We present 10-year outcome data of patients with EGC whose rib marrow was examined for micrometastases and correlate the findings with treatment and conventional pathologic tumor staging. A total of 88 patients with localized esophagogastric tumors had radical en-bloc esophagectomy, with 47 patients receiving neoadjuvant (5-fluorouracil/cisplatin based) chemoradiotherapy (CRT) and the remainder being treated with surgery alone. Rib marrow was examined for cytokeratin-18-positive cells. Standard demographic and pathologic features were recorded and patients were followed for a mean 10.04 years. Disease recurrences and all deaths in the follow-up period were recorded. No patients were lost to follow-up. 46 EGC-related and 10 non-EGC-related deaths occurred. Multivariate Cox analysis of interaction of neoadjuvant chemotherapy, nodal status, and BMM positivity showed that the contribution of BMM to disease-specific and overall survival is significant (*P* = 0.014). There is significant interaction with neoadjvant CRT (*P* < 0.005), and lymph node positivity (*P* < 0.001) but BMM positivity contributes to increase in risk of cancer-related death in patients treated with either CRT or surgery alone. Bone marrow micrometastases detected at the time of surgery for EGC is a long-term prognostic marker. Detection is a readily available, technically noncomplex test which offers a window on the metastatic process and a refinement of pathologic staging and is worthy of routine consideration.

## Introduction

Esophagogastric cancer (EGC) is the fifth commonest cause of male cancer deaths in the 40–79 age group 1. There has been a statistically significant increase in 5-year survival between the years 1975–1977 (5%) and 2001–2007 (19%), which may be attributable to a combination of earlier detection 2, improvements in surgical technique and use of perioperative therapies including neoadjuvant or adjuvant chemoradiation and perioperative chemotherapy 3–6. Tumor stage and lymph node disease are the best predictors of outcome for patients with resectable tumors 7,8, but traditional pathologic staging is suboptimal with some node negative patients having poor survival and some node-positive patients surviving longer than expected. Bone marrow micrometastases (BMM) provide a window on the metastatic process 9,10, indicate presence of minimal residual disease in potentially curative resections, and are independently prognostic in several solid tumors including lung 11,12, colorectal 13, and breast cancer 14–17. Several groups have attempted to improve EGC outcome prediction by refining staging using both nodal 17–19 and bone marrow minimal residual disease 20,21. We previously reported the high incidence of micrometastasis detection in bone marrow, with specimens obtained from rib marrow having a higher detection rate than iliac crest aspirates 22. Here, we present the 10-year follow-up of a cohort of prospectively studied patients with EGC in whom rib marrow was examined for micrometastases, and correlate outcome with conventional tumor staging, preoperative treatment, and presence of BMM.

## Patients and Methods

### Patients

Patients (*n* = 88) were prospectively recruited from two tertiary referral centers in Cork and Dublin between August 1996 and February 2002. Each had a localized esophagogastric tumor and was fit for curative surgery, without evidence of distant metastatic disease on clinical staging which as described previously 9 included laparoscopy, bronchoscopy, and computed tomography of chest and abdomen. Preoperative endoscopic ultrasound was not standard practice at that time. Informed consent was obtained for marrow analysis and the study received ethical approval from the clinical research ethics committees of the participating hospitals.

In light of previous published work 3 and a change in treatment policy, 47 of 88 patients received neoadjuvant chemoradiotherapy (CRT) prior to surgery. The neoadjuvant regimen included two cycles of 5-fluorouracil 1000 mg/m^2^ for 5 days plus cisplatin 75 mg/m^2^ on day one, with concurrent radiotherapy (40 Gy in 15 fractions). Exclusion criteria for this study were: a history of a previous tumor; patient refusal of surgery; rapid progression of disease postdiagnosis precluding surgery; and noncompletion of CRT regimen.

### Rib marrow immunohistochemistry

Posterior rib segments were excised at time of primary tumor resection prior to tumor manipulation, to facilitate rib retraction. Rib segments were processed as described previously 22. Briefly, resected rib segments were placed in citrated serum-free Dulbecco’s modified Eagle culture medium to prevent coagulation. In the laboratory, marrow was flushed from the rib segment using culture medium and fresh marrow aliquots were fixed for immunohistochemistry by dropwise addition with gentle shaking into cold 70% ethanol. The fixed sample was enriched for mononuclear cells by Ficoll-Hypaque density gradient centrifugation. Mononuclear cells (10^6^) were cytospun onto a glass slide and stained using a monoclonal anti-cytokeratin-18 antibody (Sigma-Aldrich, St. Louis, MO), and visualized using the alkaline phosphatase anti-alkaline phosphatase (APAAP) technique 6,20,21. Positive cells were detected by light microscopy. Any number of nucleated cytokeratin staining cells was taken as a positive result.

### Follow-up

Standard demographic and pathologic parameters were recorded and patients were followed until death or a mean 10.04 years, in a combination of outpatient, inpatient, and primary care settings. All disease recurrences and all deaths (including peri- and postoperative deaths) in the follow-up period were recorded. Positive recurrence included either distant or locoregional disease, and clinical end points were measured from date of primary tumor resection. Primary end points were disease-specific survival defined as time from surgery to death from EGC, and overall survival defined as time from surgery to death from any cause.

### Statistical analysis

*T*-tests and chi-squared tests were used for univariate analyses with continuous and categorical variables. The cumulative survival rates for patient groups were calculated by using Kaplan–Meier methods. Overall survival and disease-specific survival were considered. Univariate survival analysis was evaluated using log-rank tests. Survival was also evaluated using multivariate Cox regression as implemented in the R statistical package 23. Factors included in the multivariate model were: BMM; lymph node metastases; and neoadjuvant CRT. Factors were dichotomized. Possible interactions between factors were analyzed. A model for risk of death as a function of the factors was constructed using cross-validation and Akaike information criterion (AIC) 24 for model selection. Significance was assessed at the 5% level.

## Results

### Demographics and tumor characteristics

The 88 patients included 66 men and 22 women, mean age 60.5 years (range 41–81). Tumors comprised 66 adenocarcinomas (including 1 adenosquamous carcinoma) and 22 squamous cancers. Forty-seven patients received neoadjuvant CRT followed by surgical resection (CRT) with 41 treated with surgery only. Pathology showed complete response to neoadjuvant treatment in 10 of 47 (21.3%) CRT cases. Lymph node-positive disease was identified in 26 of 47 (55%) patients who received CRT, and 32 of 41 (78%) patients treated with surgery alone. Cytokeratin-positive cells within the bone marrow were detected in 47 of 88 cases (53%). The rate of micrometastasis detection with respect to tumor stage and neoadjuvant treatment is shown in Table1: no significant difference was identified between BMM-positive and BMM-negative groups for the clinical and pathologic parameters examined.

**Table 1:** Patient and tumor characteristics with respect to micrometastasis (MM) detection

	Surgery alone (*n* = 41)			Chemoradiation + surgery (*n* = 47)		
	MM positive *N* = 27 (%)	MM negative *N* = 14 (%)	*P*-value	MM positive *N* = 20 (%)	MM negative *N* = 27 (%)	*P*-value
Mean age (years)	61.6	61.5	N.S.	60.1	58.1	N.S.
Gender						
Male	18 (67)	12 (86)	0.35	14 (70)	22 (81)	0.56
Female	9 (33)	2 (14)		6 (30)	5 (19)	
Histologic type						
Adenocarcinoma	20 (74)	12 (86)	0.64	12 (60)	22 (81)	0.19
Squamous	7 (26)	2 (14)		8 (40)	5 (19)	
Nodal status						
Node negative	6 (22)	3 (21)	1.00	7 (35)	13 (48)	0.55
Node positive	21 (78)	11 (79)		13 (65)	14 (52)	
Stage						
0–1	1 (4)	2 (14)	0.55	5 (25)	7 (26)	1.00
2–3	26 (96)	12 (86)		15 (75)	27 (74)	

### Outcomes at 10 years

No patients were lost to follow-up. Overall survival at 10 years was 23.9%, with 21 patients (13 CRT, 8 surgery alone) alive and tumor free. There were 46 disease recurrences (52.3%, 23 CRT, 23 surgery alone) resulting in death and one disease recurrence (skin) with the patient still alive at last encounter. There were 11 peri/postoperative deaths (12.5%, 6 CRT, 5 surgery alone) at median 33 days (range 5–117 days) as follows: one disseminated intravascular coagulation; one acute respiratory distress syndrome; one postoperative complication, not otherwise specified; eight sepsis/multi-organ failure. There were ten non-EGC deaths (11.4%, 5 CRT, 5 surgery alone): four attributed to second primary tumors; two esophageal bleeds without evidence of tumor; one due to pneumonia; and three without identified cause and no evidence of tumor. The relationship between micrometastasis status and outcome is shown in Table2.

**Table 2:** Outcome at 10-years in relation to treatment and micrometastasis

	Alive	BMM positive	All deaths (OS)	BMM positive	OGC deaths (DSS)	BMM positive	*P*
Surgery, *n* = 41 (%)	8 (20)	3/8 (37%)	33 (80)	24/33 (73%)	23 (56)	17/23 (74%)	0.02
CRT + surgery, *n* = 47 (%)	13 (28)	6/13 (46%)	34 (72)	14/34 (41%)	23 (49)	10/23 (43%)	N.S.
Total, *n* = 88 (%)	21 (24)	9/21 (43%)	67 (76)	38/67 (57%)	46 (52)	27/46 (59%)	N.S.

### Disease-specific and overall survival

**Figure 1:**
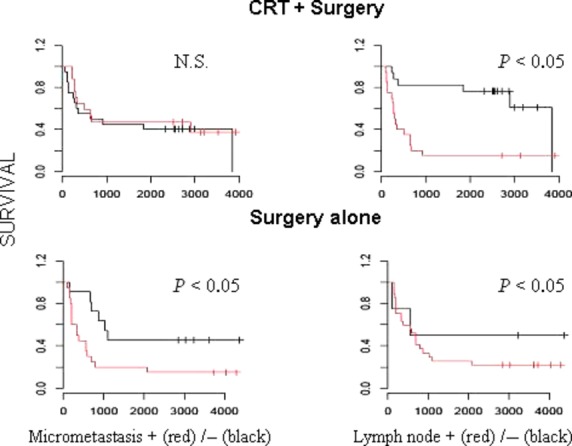
Disease-specific survival with respect to node and micrometastasis status in chemoradiotherapy (CRT) and surgery alone groups: univariate analysis shows lymph node positivity (red) separates low- and high-risk groups in both patient groups; bone marrow micrometastasis positivity (red) separates low- and high-risk groups in surgery alone but not CRT patients.

**Figure 2:**
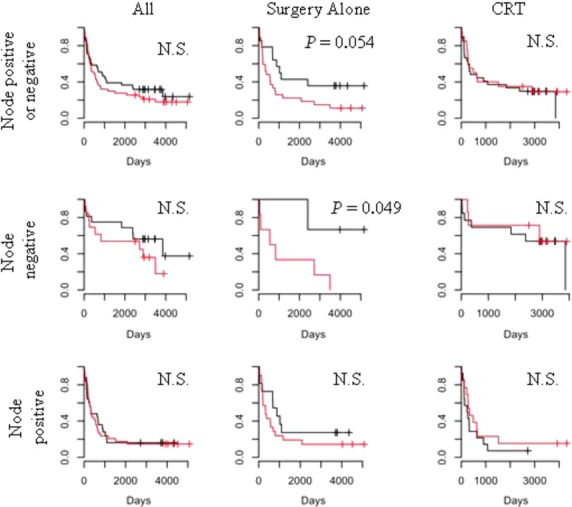
Overall (all-cause) survival, Kaplan–Meier curves: red— bone marrow micrometastases (BMM) positive; black— BMM negative.

Kaplan–Meier survival curves demonstrate that bone marrow micrometastasis positivity is a significant predictor of poorer disease-specific survival among surgery alone but not CRT patients (Fig.1), with lymph node positivity differentiating low and high-risk groups in both CRT and surgery alone patients as expected (Fig.1). Univariate analysis confirmed lymph node positivity is significantly associated with disease-specific survival (Table3). When all deaths were considered, BMM positivity separated low- and high-risk populations in node negative and approached significance in node-positive surgery alone patients (Fig.2), but was not significantly correlated with overall survival in CRT patients. Univariate analysis again confirmed lymph node positivity is significantly associated with overall survival (Table4).

**Table 3:** Univariate analysis: disease-free survival

Variable	HR	95% CI	*P*-value
Cell type			
Squamous	1.09	0.64–1.87	0.75
Adenocarcinoma			
Nodal status			
Negative			
Positive	2.32	1.33–4.06	0.003
Micromet status			
Negative			
Positive	1.22	0.75–1.98	0.42
Treatment			
SO			
CRT	0.91	0.56–1.47	0.69

**Table 4:** Univariate analysis: overall survival

Variable	HR	95% CI	*P*-value
Cell type			
Squamous	1.12	0.65–1.93	0.68
Adenocarcinoma			
Nodal status			
Negative			
Positive	2.30	1.31–4.02	0.004
Micromet status			
Negative			
Positive	1.24	0.76–2.01	0.39
Treatment			
SO			
CRT	0.94	0.58–1.52	0.80

### Multivariate analysis

**Figure 3:**
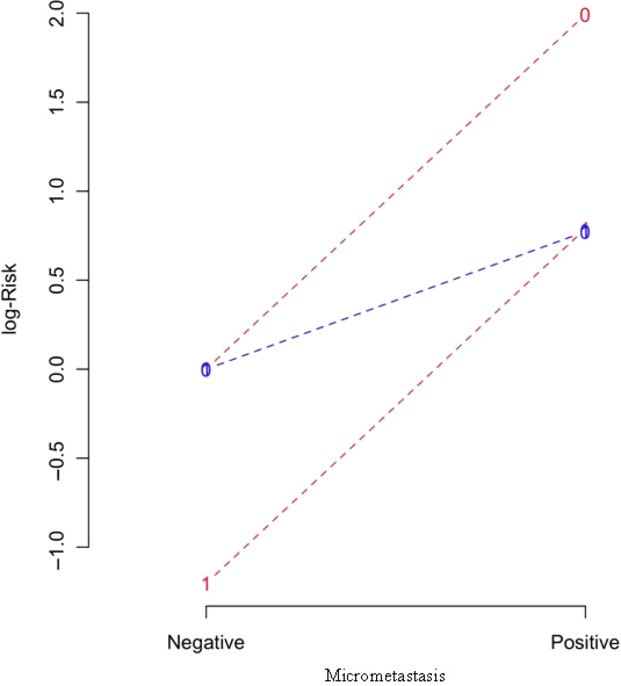
AIC model analysis of interaction between bone marrow micrometastasis and nodal status in risk of disease-specific survival: blue lines— surgery alone, red lines—chemoradiotherapy (CRT) + surgery; 0—node negative, 1—node positive. The contribution to risk of Esophagogastric cancer (EGC)-related death is increased with bone marrow micrometastases (BMM) positivity in both node-positive and node-negative surgery alone and CRT patients.

Multivariate Cox analysis of interaction of neoadjuvant chemotherapy, nodal status and BMM positivity in contributing to survival risk is shown in Tables5 and 6. The overall contribution of micrometastasis positivity to disease-specific and overall survival is significant (*P* = 0.014), but there is significant interaction with neoadjuvant CRT (*P* < 0.005), and lymph node positivity (*P* < 0.001). The AIC analysis of the interaction between risk factors is illustrated graphically in Figure3: BMM positivity contributes to increase in risk of cancer-related death in patients treated with surgery alone but also in those who received CRT).

**Table 5:** Cox’s regression (multivariate) analysis: disease-free survival

Variable	HR	95% CI	*P*-value
Micromet positive	2.17	1.17–4.00	0.014
Node and CRT positive	0.30	0.13–0.68	0.004
Micromet and CRT positive	3.24	1.70–6.20	0.0003

**Table 6:** Cox’s regression (multivariate) analysis: overall survival

Variable	HR	95% CI	*P*-value
Micromet positive	2.17	1.17–4.02	0.014
Node and CRT positive	0.30	0.13–0.69	0.005
Micromet and CRT positive	3.39	1.77–6.49	0.0002

### Effect of chemotherapy

While examination of neoadjuvant treatment efficacy was not an aim of this study some treatment-related effects were evident. A higher percentage of CRT patients were alive at 10 years (28%) compared to surgery alone patients (20%), in keeping with previous data indicating a survival advantage with this chemoradiation strategy 3. Response to neoadjuvant therapy was an important prognostic factor in patients treated with CRT: seven of eleven patients (64%) achieving complete pathologic response were alive at 10 years. In addition 50% of patients who received CRT who had uninvolved nodes at surgery were alive at 10 years versus 22% of patients who had surgery alone with uninvolved nodes. None of these effects reached statistical significance.

## Discussion

Over the last two decades several investigators have examined the prognostic significance of BMM in patients with EGC using a variety of antibodies, epitopes, and detection methods 21,22,25–27, but long-term outcome data are scant. This study represents, to our knowledge, the largest series of patients with EGC in whom BMM is correlated with 10-year follow-up data. Studies to date have all reported a proportion of patients with rare cells detected in marrow exhibiting epithelial cell phenotype. The hypothesis that such cells have potential biologic significance is attractive given the high rate of progression in patients with localized disease, including cases with pathologically proven complete pathologic response of the primary tumor to neoadjuvant chemoradiation. Marrow micrometastases provide insight into the metastatic process, and reflect synchronous minimal residual disease present in other tissues such as liver and lung, and BMM have been shown to be independently prognostically significant in many solid tumors including lung 11,12, and breast 16.

Direct comparison of studies of BMM in EGC is complicated by the different methods employed by different groups of investigators—to date there is no accepted standard methodology for detection of BMM—with other potential confounders including sample size, shorter follow-up, and heterogeneity of neoadjuvant treatments and surgical techniques employed 28. Such differences may explain why many studies of patients with upper gastrointestinal cancers have found BMM or surrogate markers such as CK19 mRNA to be independently prognostically significant 20,25,29,30 but others have not 21,31. Our method used cytokeratin immunohistochemistry which is a standard technique in routine use in most pathology laboratories, commonly employed in detection of metastatic carcinoma, and rib marrow was sampled as we have previously shown that it is superior to pooled bilateral iliac crest aspirates for BMM detection 22, a finding that has been replicated in another center 32.

The rate of EGC-related death at 10 years of 52% in this study and in our previous report of viable BMM present up to 12 months postoperatively 9 attest to the biologic potential of BMM as true residual malignant disease. Recent experimental evidence supports a model whereby tumors are sustained by a minor population of cells which have stem cell characteristics of self-renewal 33. Tumor stem cells have been demonstrated in hematological malignancies 34 and several solid tumors including breast 35 and colon 36,37, and some preliminary published work supports BMM-expression of tumor stem cell markers being an independent prognosticator in breast cancer 38. There are similar implications for circulating tumor cells in peripheral blood, investigation of which reflects ever-improving technical ability to detect these cells and allows quantitative estimation of tumor cell burden. Circulating tumor cells have been shown to be prognostic in advanced breast cancer 39, although no clinical management implications have resulted as yet. Efforts to identify BMM or circulating tumor cells which harbor stem cell markers are important but do not negate BMM as a staging biomarker which (when absent) may save patients from nonbeneficial adjuvant therapies, particularly in patients with apparently early stage disease where perioperative therapies are being considered. The AIC risk interaction model analysis highlights the contribution made by BMM to increase in risk across all patient groups, and clinical trials are currently enrolling for patients with advanced breast cancer where detection of circulating tumor cells is used to direct preoperative and systemic therapy (NIH Protocol ids 08128, CA 111359, NCT01048918).

In summary, the majority of patients undergoing potentially curative resection for EGC die of systemic disease which is present at time of surgery and may be detected in rib marrow. Detection of BMM in rib marrow is associated with increased risk of cancer-related death at 10 years in all patients and is independently prognostically significant in patients who do not receive CRT prior to surgery. Analysis of rib marrow for micrometastases is a useful supplementary strategy in refining the staging of patients with apparently early stage disease who are proceeding to surgery.

## Conflict of Interest

None declared.
